# Novel time-domain NMR-based traits for rapid, label-free Olive oils profiling

**DOI:** 10.1038/s41538-022-00173-z

**Published:** 2022-12-13

**Authors:** Vasco Rafael dos Santos, Victor Goncalves, Peishan Deng, Ana Cristina Ribeiro, Mariana Maia Teigao, Bárbara Dias, Inês Mendes Pinto, Juan Gallo, Weng Kung Peng

**Affiliations:** 1 University of Minho, Braga, 4704-553 Portugal; 2 International Iberian Nanotechnology Laboratory, Braga, 4715-330 Portugal; 3 Songshan Lake Materials Laboratory, 523-808 Dongguan, China; 4 Instituto de Investigação e Inovação em Saúde, Universidade do Porto, Porto, Portugal

**Keywords:** Solution-state NMR, Agriculture, Biomedical engineering, Fatty acids

## Abstract

Olive oil is one of the oldest and essential edible oils in the market. The classification of olive oils (e.g. extra virgin, virgin, refined) is often influenced by factors ranging from its complex inherent physiochemical properties (e.g. fatty acid profiles) to the undisclosed manufacturing processes. Therefore, olive oils have been the target of adulteration due to its profitable margin. In this work, we demonstrate that multi-parametric time-domain NMR relaxometry can be used to rapidly (in minutes) identify and classify olive oils in label-free and non-destructive manner. The subtle differences in molecular microenvironment of the olive oils induce substantial changes in the relaxation mechanism in the time-domain NMR regime. We demonstrated that the proposed NMR-relaxation based detection (AUC = 0.95) is far more sensitive and specific than the current gold-standards in the field i.e. near-infrared spectroscopy (AUC = 0.84) and Ultraviolet-visible spectroscopy (AUC = 0.73), respectively. We further show that, albeit the inherent complexity of olive plant natural phenotypic variations, the proposed NMR-relaxation based traits may be a viable mean (AUC = 0.71) in tracing the regions of origin for olive trees, in agreement with their geographical orientation.

## Introduction

**Fig. 1:**
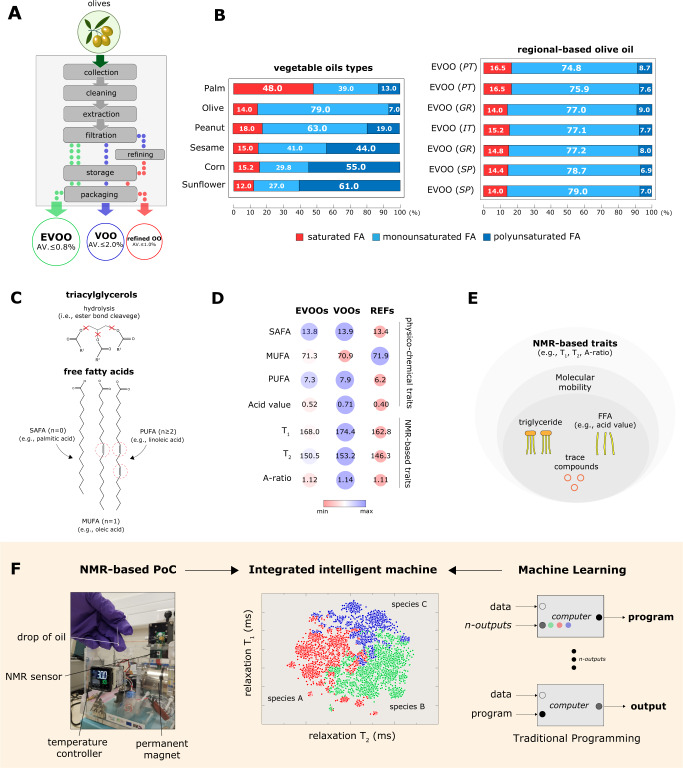
Identification and classification of Olive oils using home-built NMR-based PoC. **A** Olive oil production process. The mechanical processes (e.g. cold press) in the extraction of olive oils, in particularly in the separation phase (e.g. filtration, refining) play a major role in preserving the amount of free fatty acids in OOs, and thus in the final grading of the product (e.g. EVOOs, VOOs, and refined OO). EVOO must be obtained using exclusively mechanical extraction procedures and preserving an acid value (AV) of less than 0.8%. On the other hand, acid value of VOO and refined OOs must be below 2.0% and 1.0%, respectively. **B** In OOs, fatty acids are predominantly in the form of triacylglycerols and are defined by their saturation level (e.g. saturated, monounsaturated, polyunsaturated). FA concentration varies greatly based on the type of vegetable oil and the regions of origin of the starting products. OOs are dominated by oleic acid (monounsaturated FA), similarly to peanut oil. Palm oils are saturated FA dominant, in contrast to sunflower and corn oils which are polyunsaturated FA dominant. **C** Olive oil composition is dominated by triacylglycerols (e.g. triolein) with free fatty acids and ‘trace’ compounds (e.g. vitamin E, and other bioactive molecules) in smaller amounts. Fatty acids are characterized by their saturation level (n) and by the hydrocarbon chain properties (mainly length). For n = 0, FAs are known as SAFAs (saturated FAs). For n = 1 and n > 1, FAs are known as MUFAs (monounsaturated) and PUFAs (polyunsaturated), respectively. An increase in free fatty acid pool (e.g. acidity) occurs due to the hydrolysis of triacylglycerols esters. **D** A bird-eye view of the relationship between the NMR-based traits, physiochemical properties, and OOs grading (EVOO, VOO, and refined OOs). The classification of OOs is influenced by the FA and FFA profiles, among other factors. **E** NMR-based traits originate on the relaxation dynamics of nuclear spins of proton nuclei due to the composite effect of triacylglycerols, free fatty acids, trace compounds, and the overall environment of the protons. **F** The concept of using integrated intelligent machine as proposed in this work. The developed NMR-based PoC consists of a portable commercial console, home-built detection circuit coil, and a palm-sized permanent magnet (B = 0.5 T). For high-throughput analysis, a microcapillary tube designed to be slotted into the NMR detection coil is used to store minute sample (e.g. a single drop of oil). The entire assay completes in less than 5 min. The NMR measurements were carried out in single blinded manner on each oil.

Olive oil (OO) is one of the oldest and essential edible oils commercially traded in the history of mankind. Olive oils are commonly classified into extra-virgin olive oils (EVOOs), virgin olive oils (VOOs) or mixed with refined olive oils (refined OOs) (Fig. 1A), depending on among other factors, its fatty acids (FA) profiles and the trace compounds (e.g. concentration of free fatty acids (FFA) or acid value (AV)^1–4^, phenolic compounds^5^). FAs are predominantly defined by its saturation levels (e.g. saturated fatty acids (SAFA), monounsaturated fatty acids (MUFA) and polyunsaturated fatty acids (PUFA)) (Fig. 1B). The FFA content is influenced by a number of phytosanitary factors and extraction processes^6–8^. As a consequent of variation in processing (e.g. poor olive quality or inadequate extraction process), triacylglycerols structural breakdown may occurs (due to for example high temperature and moisture induced hydrolysis^9^), resulted in an increment in the final acidity of the oils^3,4^ (Fig. 1C, D).

The high demand of OO comes from its multiple nutritional benefits and its irreplaceable organoleptic properties^10,11^. Olive oil is by far one of the most frequently adulterated food products due to its high customer appeal and large profitable margin^12,13^. The highly desired and expensive EVOO is frequently diluted with cheaper adulterated oils, leading to indirect economic consequences and health concerns. Hence, olive oil has been the subject of rigorous quality regulations, with its standardization characteristics set amid tight legislation.

Laboratory-based methods, such as chromatography^13–16^, spectroscopy^12,17–21^, or DNA analysis^13,22,23^, have been extensively developed to reduce the cases of adulteration. Nuclear magnetic resonance (NMR) spectroscopy in the high-field frequency domain has also been proposed to be an effective method on the detection of authentication, quality control, and adulteration of the oils. High-field NMR, however, has a number of drawbacks, such as the requirement of large, dedicated laboratory facilities with costly cryogenic cooling gases, complicated pre-analysis steps, and the need of a highly specialized workforce^17,24,25^. None of the above-mentioned detection methods are simple to use, require minimal sample preparation, nor present short turn-around time.

We have recently demonstrated that two-dimensional time-domain NMR can be used to classify edible oils based on their physiochemical composition (e.g. saturation levels) with much higher accuracy than the conventional methods^26^. The low-field NMR-based point-of-care (PoC)^27–32^analysis is based on pairing the longitudinal (T_1_) and transversal (T_2_) relaxation times, which improves the sensitivity and specificity of the detection significantly. It works on the rationale that accumulative characteristics of each dimensionality form a specific and unique signature, in a way similar to the radiomics technique developed in the field of radiology.

In this work, we demonstrate that NMR-based phenotypic traits in the time-domain (at molecular level) can be used for classifying the OOs. Using just a single droplet, we demonstrated that using benchtop sized NMR^33–35^, olive oils can be rapidly classified (into EVOOs, VOOs or refined OOs) in non-destructive manner (i.e. label-free or without sample pre-treatment). The subtle differences in physiochemical composition and molecular microenvironment of the olive oils induce substantial changes in the relaxation mechanism in the time-domain NMR regime (Fig. 1E, F). With the aid of machine learning, the sensitivity and specificity of the detection were shown to have AUC = 0.95 using T_1_ relaxation and T_2_ relaxation, much higher than current gold-standards, the near-infrared spectroscopy (NIRS, AUC = 0.84) and Ultraviolet-Visible spectroscopy (UV-Vis, AUC = 0.73) (Table 1), and much better performance in the identification of regions of origin (Table 2). In addition, the proposed NMR-based detection methods were much cheaper per assay, user-friendly, and can be used at point-of-detection (Table 3). This work demonstrated the spirit of combining the (old-fashioned) machine with the (new-wave) of machine learning, to produce an ′intelligent machine′^30,36,37^, an attractive scientific solution for the food science community.

**Table 1:** Classification of olive oils using the Receiver Operating Characteristic analysis.

techniques	Model	AUC	CA	F1	Precision	Recall
UV-VIS	kNN	0.898	0.833	0.833	0.835	0.833
(*λ* = 415 nm)	Logistic Regression	0.444	0.417	0.37	0.333	0.417
	Naive Bayes	0.615	0.472	0.479	0.495	0.472
	Neural Network	0.762	0.667	0.663	0.672	0.667
	Random Forest	0.937	0.778	0.781	0.79	0.778
	Average	0.731	0.633	0.625	0.625	0.633
NIRS	kNN	1.000	1.000	1.000	1.000	1.000
(*λ* = 670 nm)	Logistic Regression	0.542	0.417	0.362	0.333	0.417
	Naive Bayes	0.771	0.75	0.743	0.778	0.75
	Neural Network	0.875	0.833	0.822	0.889	0.833
	Random Forest	1.000	1.000	1.000	1.000	1.000
	Average	0.838	0.800	0.785	0.800	0.800
NMR-based traits	kNN	0.974	0.889	0.889	0.889	0.889
	Logistic Regression	0.984	0.889	0.889	0.889	0.889
	Naive Bayes	0.95	0.861	0.864	0.878	0.861
	Neural Network	0.918	0.889	0.889	0.889	0.889
	Random Forest	0.919	0.833	0.831	0.834	0.833
	Average	0.949	0.872	0.872	0.876	0.872

Area Under the Curve (range between 0 and 1) of the various supervised models evaluated to predict the oil types. Models were validated using Leave-one-out method with averaged NMR-based traits (e.g. T_1_ relaxation, T_*2*_ relaxation, and A-ratio). The wavelength (*λ*) used for UV-Vis spectroscopy (at 415 nm) and NIRS (at 670 nm) was chosen upon the region of highest peaks resolution. Confusion matrix of individual models is displayed in Supplementary Fig. 7.

**Table 2:** Receiver Operating Characteristic analysis for regions of origin.

techniques	Model	AUC	CA	F1	Precision	Recall
UV-VIS	kNN	0.856	0.667	0.646	0.679	0.667
(*λ* = 670 nm)	Logistic Regression	0.403	0.179	0.203	0.274	0.179
	Naive Bayes	0.674	0.385	0.387	0.422	0.385
	Neural Network	0.751	0.641	0.62	0.684	0.641
	Random Forest	0.786	0.694	0.685	0.704	0.694
	Average	0.694	0.513	0.508	0.553	0.513
NIRS	kNN	0.856	0.667	0.646	0.679	0.667
(*λ* = 670 nm)	Logistic Regression	0.43	0.179	0.131	0.127	0.179
	Naive Bayes	0.687	0.385	0.383	0.382	0.385
	Neural Network	0.753	0.436	0.433	0.456	0.436
	Random Forest	0.752	0.641	0.625	0.635	0.641
	Average	0.696	0.462	0.444	0.456	0.462
NMR-based traits	kNN	0.718	0.538	0.539	0.541	0.538
	Logistic Regression	0.658	0.433	0.365	0.331	0.433
	Naive Bayes	0.667	0.433	0.383	0.348	0.433
	Neural Network	0.788	0.576	0.561	0.571	0.576
	Random Forest	0.699	0.500	0.497	0.497	0.500
	Average	0.706	0.496	0.469	0.458	0.496

Area Under the Curve (range between 0 and 1) of the various supervised models evaluated to predict OO origin. Models were validated using Leave-one-out method with NMR-based traits (e.g. T_1_ relaxation, T_*2*_ relaxation, and A-ratio). The wavelength (*λ*) used for UV-Vis spectroscopy and NIRS was 670 nm (chosen under the region with highest resolution). Confusion matrixes of individual models are displayed in Supplementary Fig. 8.

**Table 3:** Qualitative performance of the NMR-based PoC against conventional methods (UV-Vis, NIR).

features	Integrated NMR-based PoC	UV-VIS, NIR Spectrometry
Sensitivity	Very high	Medium/high
Specificity	Very high	Medium/high
ROC (AUC)	(0.95)	(0.73, 0.84)
Extensive experience	Not required	Not required
Time to results	Minutes	Minutes
Sample processing	Nil (no solvents needed)	Need specific solvents, nil
Price per assay	Ultra-cheap	Expensive (cuvettes, solvents)
Equipment size	Point-of-care testing	Benchtop

SWOT-like analysis between the state-of-the-art technologies (e.g. Near-Infrared spectroscopy and UV-Vis (Supplementary Figs. 9–10)) versus the integrated intelligent machine proposed in this work (machine learning assisted NMR-based PoC).

## Results

### Rapid identification and characterization of olive oils with NMR-based PoC

**Fig. 2:**
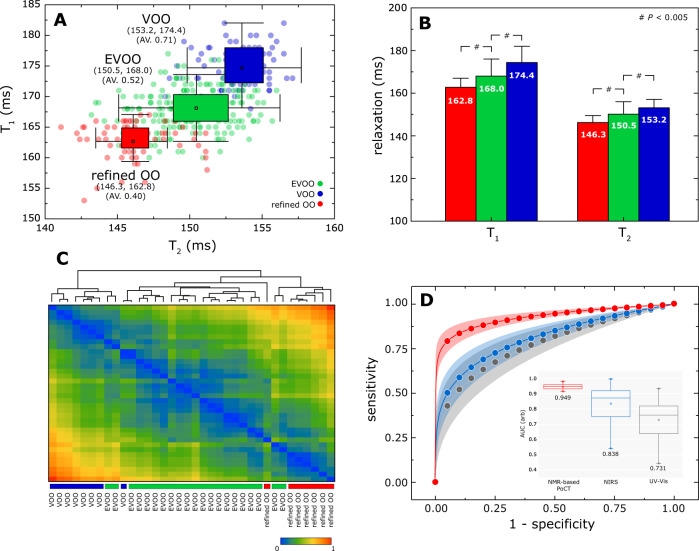
Rapid identification and characterization of olive oil using NMR-based traits. **A** Two-dimensional mapping of EVOOs (green), VOOs (blue), and refined OO (red) in the T_1_-T_2_ magnetic phase diagram. A wide variety of Olive oils (i.e. 21 EVOOs, 8 VOOs, and 7 refined OOs) commercially available from different manufacturers were purchased off-the-shelf in Braga, Portugal or otherwise through online platforms. Each data point represents one sampling with a total 360 samplings collected. The mean T_1_ and T_2_ values and acid value were denoted below. **B** Average T_1_ and T_2_ relaxation times (ms) for the different types of OOs. The statistical analysis of the data was calculated using unpaired two-tailed Student *t*-tests (*P* < 0.005) (details in Supplementary Fig. 1). **C** Rapid classification of OOs using the NMR-based traits in the form of clustering analysis. This hierarchical clustering was constructed based on the Euclidean distance between the averaged measures per sample (details in Supplementary Table 1). Their quantitative linkages (e.g. inter- and intra-cluster similarity) are shown as a heatmap. **D** The ROC curves for NMR-based traits (red), NIRS (blue), and UV-Vis (grey) calculated from a number of supervised models (details in Supplementary Fig. 2). The error zone were 99% of the confidence band.

In order to demonstrate the industrial applications, we use the proposed technique to validate the authenticity of EVOO from VOOs and refined OO (Fig. 2). The relaxometry measurements and acidity determination (details in Methods) were performed on thirty-six types of OOs (i.e. 21 EVOOs, 8 VOOs, and 7 refined OOs,) without disclosing the manufacturers label and country of origin. For each sample, the relaxation measurements were carried out using five different samplings, with the refined OO was performed as control experiment.

A two-dimensional map T_1_-T_2_ magnetic state diagram was used to enumerate the object clustering based on the composite intrinsic relaxation properties of the oils, thereof, forming a calibration standard for the (EVOOs, VOOs, refined OOs), and (150.5 ms, 168.0 ms), (153.2 ms, 174.4 ms), (146.3 ms, 162.8 ms), respectively (Fig. 2A and details in Supplementary Fig. 1).The oil types were significantly clustered (*P* < 0.005) indicate that the intra-variation samplings were much smaller than the inter-variation of the OOs (Fig. 2B).The details breakdown for each commercial brand is shown in heatmap (Fig. 2C).In addition, the Receiver Operating Characteristics (ROC) analysis (Fig. 2D and Supplementary Fig. 2) indicated that relaxometry measures have excellent detection sensitivity and specificity with Area Under the Curve (AUC) of 0.95 as compared to its counterparts NIRS (0.84) and UV-Vis (0.73), respectively (Table 1).

### Identification of OO based on the regions of origin

**Fig. 3:**
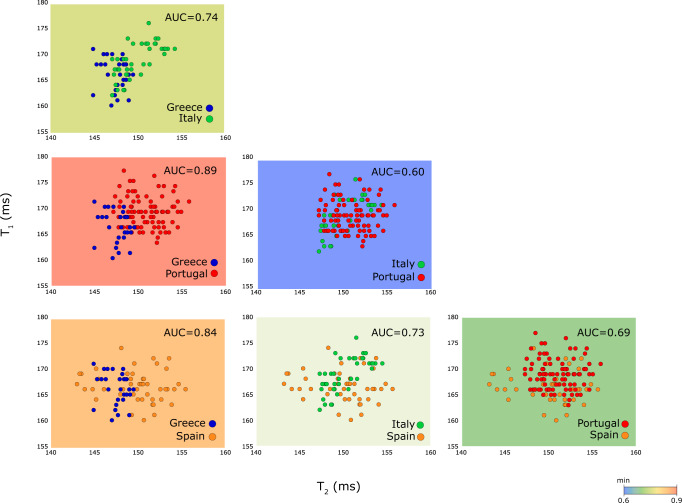
Identification of the regions of origin of OO. EVOO samples were studied based on their regions of origin. Off-the-shelf EVOO samples originated from different European regions (i.e. 9 Portugal, 5 Spain, 4 Italy, 3 Greece) according to their labelling. Pair-wise two-dimensional mapping of EVOOs origin in the T_1_-T_2_ magnetic state diagram, according to unpaired two-tailed Student *t*-tests (details in Supplementary Fig. 3). The sensitivity and specificity of each region were calculated using receiver operating characteristic (ROC). The substantially high AUC, ranging from 0.6 to 0.9 of each pair/wise region were evaluated.

We demonstrated the feasibility of using the proposed NMR analyses in identification of production based on their countries (or regions) of origin. Apart from the genotypic variation, the variation in phenotypic traits is governed by number of factors, such as migration drift (e.g. diversification and domestication events)^38^, and abiotic stress (e.g. local climate, soil conditions)^39,40^. For the identification of the regions of origin for OO, a matrix of data subsets, encompasses four different regions taken from the European regions (i.e. 3 Greece, 4 Italy, 9 Portugal, and 5 Spain) were enumerated using two-dimensional T_1_-T_2_ magnetic state diagram (Fig. 3) and the details of each oil variations (details in Supplementary Fig. 3, and Supplementary Table 1).

**Fig. 4:**
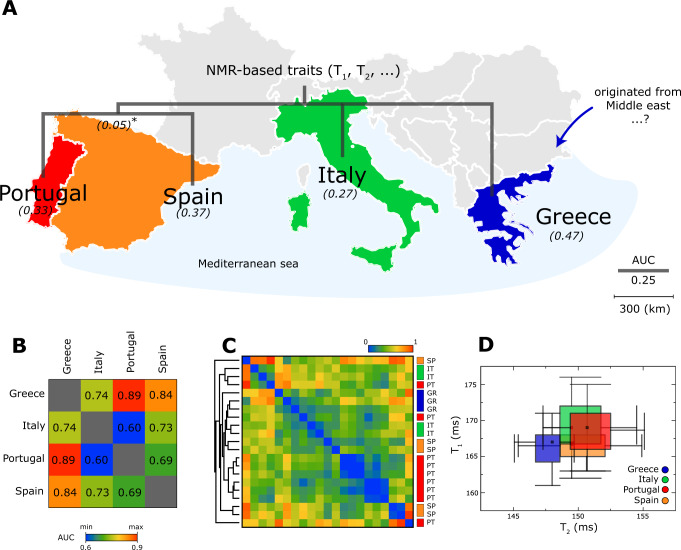
NMR-based traits in identification of the regions of origin. **A** The NMR-based phylogenetic tree was built using the AUC distance matrix (details in Fig. 3) using neighbour joining algorithm (Supplementary Fig. 6) which splits the NMR-based traits into three main regions (i.e. Iberian, Italy, Greece). Iberian are Portugal and Spain which shared the same land border. The proposed NMR-relaxation based traits (legends of AUC is 0.25 in vertical) in agreement with their geographical orientation (shown in legend of 300 km per cm). Higher similarities are expected to be found species that are closely related. Neighbouring countries are expected to have higher species exchange and genes flow due to their geographical proximity. Similarities fade away with, for example, geographical distance. **B** A summary of the AUCs between countries evaluated using ROC analysis (in details in Fig. 3). **C** The detailed analysis of each commercial brands represented in the form of heatmap. Hierarchical clustering was constructed based on the Euclidean distance (between T_1_ relaxation, T_2_ relaxation, and A-ratio) of the averaged measurement per sample (Supplementary Table 1). **D** T_1_-T_2_ magnetic state diagram in the forms of box plots for the regions of origin.

The mean T_1_ relaxation times of (166.3, 166.7, 168.9, and 168.9) ms, and for T_2_ relaxation times of (147.7, 150.1, 150.2, and 151.0) ms for (Greece, Spain, Italy, Portugal), respectively (Fig. 3, and details in Supplementary Fig. 3). The regional-based identification for NMR technique is AUC = 0.71, much higher or comparable to NIRS (AUC = 0.70) and UV-Vis (AUC = 0.69) (details in Table 2). Interestingly, when a pair-wise comparison matrix (i.e. pair-wise ROC-AUC evaluation) is employed using NMR-based traits (e.g. T_1_, T_2_, A-ratio) it resembles the geographical orientation (Fig. 4A). For example, Greece-Italy (AUC = 0.74), Greece-Spain (AUC = 0.84), and Greece-Portugal (AUC = 0.89) shown as a heatmap (Fig. 4B). The Iberian region (i.e. Spain-Portugal) and Italy-Greece displayed stronger similarities with AUCs of (0.69, 0.74), respectively. This is to be expected as neighbouring countries are expected to have much higher of species exchange due to its proximity in geographical location. The details of each oils purchased displayed a unique information on their location (Fig. 4C and D).

### Limit of detection of NMR-based traits technique

We evaluated the limit-of-detection of NMR-based traits by mixing sunflower oil into a selected EVOO, to mimic the cases of adulteration. For each sample, the relaxation measurements were conducted in double using five different samplings, covering from 0% (as control) to 100% of OO in the mixed edible oil (Supplementary Fig. 4). As clearly indicated in the T_1_-T_2_ magnetic state diagram, a linear relation (r^2^ = 0.93) between NMR-based traits and the concentration of sunflower oil (PUFA-rich) reduced into EVOOs (MUFA-rich) relaxation effect becomes clearer (due to a decrease in saturation level). Therefore, the (T_2_, T_1_) coordinates were (188.3, 202.9) and (155.3, 174.6) for sunflower oils and EVOO (controls), respectively. The limit of detection for NMR-based traits were approximately (1%), were either comparable to NIRS (1%) or much better than UV-Vis spectroscopy (5%) (details in Supplementary Fig. 4).

## Discussion

We report NMR-based point-of-care technology for fast, label-free, and distinctive OO profiling and to assure its high quality, which can be used to reduce the attempts in adulteration. The NMR-based phenotypic traits represent the intrinsic molecular relaxation dynamics (or molecular mobility) due to the composite effect of the FA profiles (e.g. saturation level) and concentration of FFA (e.g. acid value). Nevertheless, despite OOs consists of predominantly the monounsaturated fat (more than 70%), we found in this work that the overall saturation levels (e.g. increasing PUFA/MUFA ratio, lower SAFA content) has profound impact on the NMR traits (details in Supplementary Fig. 5). Secondly, we observed that FFA concentration has direct effect on the NMR-based phenotypic traits. We hypothesized that, with similar mechanism i.e. the saturation levels and FFA concentration disrupts the packing^41,42^ ‘efficiency’ (i.e. weakening of Van der Walls forces) leading to a disruption in the molecular mobility and hence introducing much longer pathways for relaxations (i.e. longer T_1_ and T_2_). This is in agreement with the recent work reported by Cistola^43^.

Conventionally, chromatographic-based techniques, are extremely slow, time-consuming, require complicated multiple sample preparation steps with expensive laboratory equipment, while complicated chemometric analysis (e.g. vibrational, RAMAN spectroscopy) is required for in depth data interpretation, in comparison to the proposed NMR-based detection methods and other state-of-the-art technologies (refer to SWOT-like Table 3). The information derived from the analytical instrument represents one of the major challenges faced by food scientist during the identification and classification of pure and adulterated food samples. With the introduction of EU Protected Designation of Origin registration and equivalent in other geographical locations, rapid classification (preferably in non-destructive manner) of EVOOs will be invaluable to industry and regulatory agencies alike.

On the other hand, the proposed NMR-based technology provides rapid, precise, low-cost, label-free, and accurate analysis for grading the olive oils quality using the NMR-based phenotypic traits in the time-domain NMR. In this framework, the central hypothesis of radiomics is that it is possible to decode tissue characteristics and pathology by examining the textural features in medical images. Similarly, clustering NMR techniques work on the rationale that accumulative characteristics of each dimensionality form a specific and unique signature (‘molecular fingerprint’) is extremely powerful for rapid and accurate classification of OOs based on the NMR-based phenotypic traits. In addition, with the introduction of machine learning, it is now inexpensive to process large datasets running in almost real-time setting, opening door to intelligent machine which can make interpretation with much higher sensitivity and specificity.

## Methods

### Details and sample preparation of the OOs

OOs analyzed were cooking oils bought locally in Braga, Portugal or purchased online (e.g. international brands). The commercial brands names were disclosed (in details in Supplementary Table 2). No further processing was made before the NMR measurements and all other measurements.

### NMR measurements and parameters

The ^1^H magnetic resonance measurements of olive oils were acquired at the resonance frequency of 21.7579 MHz polarized using a portable permanent magnet (Metrolab Instruments, Switzerland), *B*_o_ = 0.5 T, using a benchtop-type console (Kea Magritek, New Zealand). A temperature controller was set to maintain the measurement chamber at 30 °C. The T_1_ relaxation and T_2_ relaxation times were acquired using standard inversion recovery (IR) and Carr-Purcell-Meiboom-Gill (CPMG) train pulse sequences, respectively. The experimental parameters used were echo time = 200 μs, number of echoes = 10,000, and signal averaging = 32. A recycle delay of 2 s was set between each experiment to provide sufficiently long time to allow all molecular spins to return to thermal equilibrium. (T_1_ relaxation, T_2_ relaxation) measurements were carried out on commercial EVOOs, VOOs and refined OOs. NMR measurements were performed blindly on each oil ten repeated times, with a total of 360 points for olive type classification, and 210 points for origin assessment. Clustering NMR methodology uses a pair of relaxation times (T_2_, T_1_) for each object (oils in this case) to construct a (pseudo) two-dimensional map (Figs. 2A and 3).

### UV-VIS and NIR measurements and detection

UV-Vis measurements were performed in a SHIMADZU UV-2550 spectrophotometer (Kyoto, Kyoto, Japan), while for NIR measurements a PerkinElmer LAMBDA 950 instrument was used. All samples were measured in matched 1 cm path length quartz or optical glass cells, running an empty cell as a reference. UV-Vis spectra were measured within 200–800 nm spectral range at 1 nm spectral resolution, while NIR, spectra were obtained within 500–2200 nm with 5 nm steps. NIR spectra spike removal algorithms^44^ were applied (cut-off = 6, threshold = 10).Every sample was measured three times and the mean values were taken as representation.

### Acid value measurements

The acid value determination was performed under the EN ISO 660:2009^45^ protocol for oleic acid quantification. Simply, 10 mL of edible oil were weighted and diluted in 20 mL of ethanol (φ = 99%,) with small amounts of phenolphthalein. Titrations with 0.1 mol/L of potassium hydroxide (KOH) were done under magnetic stirring until slight colour changes appear (and persisted for +10 s). Measures were executed twice per sample. The acid value was extrapolated from the amount of KOH required for each sample, defined as the amount of KOH required to neutralize 1 g of chemical substance, with the following formula:

1 $$w_{AV} = \frac{{56.1 \times cV}}{m}$$

where, *c* is the exact concentration of the standard KOH solution (mol/L), *V* the volume of KOH added (mL), and *m* the mass (g) of the test portion. Acidity, or the free fatty acid content, can be estimated by:

2 $$w_{{\mathrm{FFA}}} = \frac{{VcM}}{{10 \times m}} \approx 0.5 \times w_{AV}$$

wherein, *M* is the molar mass (g/mol) of the predominant fatty acid in the edible oil, in this case oleic acid (282.47 g/mol).

### Machine learning algorithm and workflows

Using statistical programming languages (e.g. Orange 3.1.2^46^ or *R*), the raw datasets were processed using supervised and unsupervised learning techniques. The machine learning algorithms were written and run on a personal laptop (Intel Core Pentium i7 CPU @ 2.70 GHz, 8.00GB RAM). Once the model in machine learning was built, all the tasks run simultaneously and completed typically in less than 1 min. Using unsupervised learning, the relationship between each object was rapidly constructed using clustering analysis (e.g. hierarchical clustering) and its quantitative linkages (e.g. inter-/intra-cluster similarity) were shown on a dendrogram and a heatmap. Supervised learning models (i.e. Neural Network, kNN, Logistic Regression, Naive Bayes, and Random Forest) were used to train the datasets and the best model with the highest accuracy was chosen to predict the object classification (e.g. oil classification) using pre-trained datasets.

### Statistical analysis

For any two groups of separation, it is considered as statistically significant when this criterion (*P* < 0.5) is achieved or otherwise denote as non-significant (n.s). The student’s unpaired *t*-test was used throughout this study. One-tailed and two-tailed were used as mentioned in the figure captions. OriginLab–Pro 8 was used to handle all the graphs plotting.

### Receiving operating characteristic

The analyses were used to evaluate the specificity and sensitivity of the diagnostic techniques. Various supervised models were used for the ROC tests. These were namely the kNN, Logistic Regression, Naïve Bayes, Neural Network, and Random Forest models. A fitting of power function *y* = *ax*^b^ were used through the study. Iterations were run with the Levenberg–Marquardt algorithm until a chi-squared tolerance of 10^−9^ was achieved. Final function AUC was compared to the real averaged AUC from all supervised models (details in Supplementary Fig. 2).

### Reporting summary

Further information on research design is available in the Nature Research Reporting Summary linked to this article.

## Supplementary information

Supplementary Information

Reporting Summary

## Data Availability

All of the datasets used in these analyses were shared in Supplementary Information or available from the corresponding authors upon request. All the raw data are shared at https://github.com/VascoRafaelSantos/OliveOil-profiling.

## Code availability

The code to reproduce the analysis and figures is available from the corresponding authors on reasonable request.
