# Supplementary material for: Novel time-domain NMR-based traits for rapid, label-free Olive oils profiling
Source: NPJ Sci Food. 2022 Dec 13;6:59. doi: 10.1038/s41538-022-00173-z (PMC9746572; doi:10.1038/s41538-022-00173-z)
Supplement: Supplementary file 1 — Supplementary Information [file 41538_2022_173_MOESM1_ESM.pdf]

Supplementary Information

Novel time-domain NMR-based traits for rapid, label-free Olive oils profiling

Vasco Rafael dos Santos[1,2,3], Victor Goncalves[2], Peishan Deng[3],
Ana Cristina Ribeiro[1,2], Mariana Maia Teigao[2], Bárbara Dias[2], Inês Mendes Pinto[2,4], Juan Gallo[2],
Weng Kung Peng [2,3]*

[1]University of Minho, Braga, 4704-553 Portugal
[2]International Iberian Nanotechnology Laboratory, Braga, 4715-330 Portugal
[3]Songshan Lake Materials Laboratory, Dongguan, 523-808 China.
[4]Instituto de Investigação e Inovação em Saúde, Universidade do Porto, Portugal

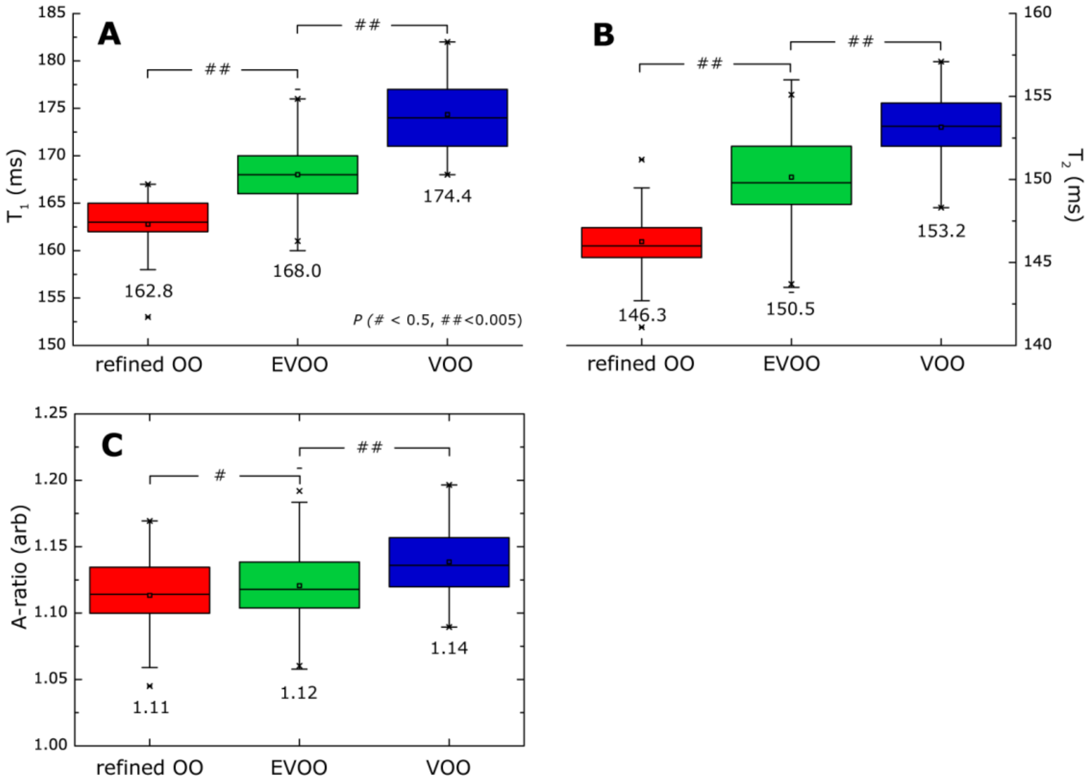

Supplementary Figure 1. Identification and classification of OO using NMR-based traits. (A) T_1 and (B) T_2 relaxations, (C) T_1/T_2 (or A-ratio) obtained for the different commercial brands (7 refined OOs, 21 EVOOs and 8 VOOs). The box plots represent 25% and 75% quantile of the entire measurements. Two tailed Student's *t*-test was used to calculate the *P*-value.

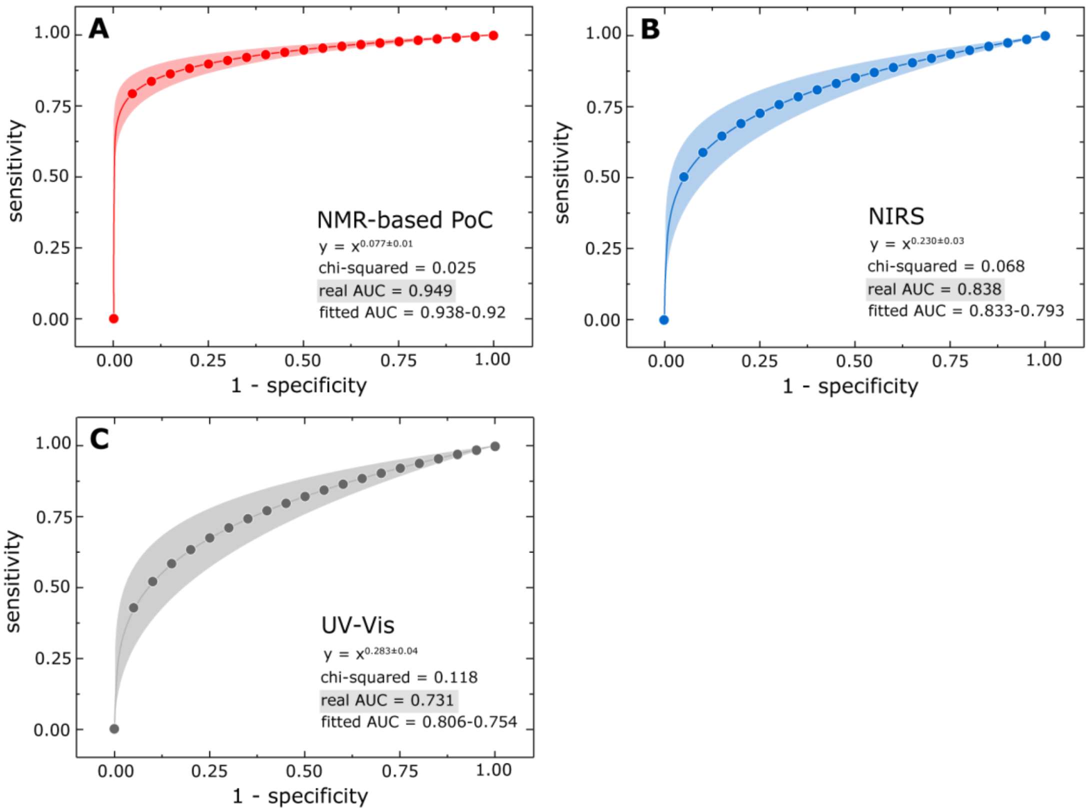

Supplementary Figure 2. Performance of OO classification as evaluated by ROC analysis. Identification and characterization of OO types (e.g., VOO, EVOO, refined OO) with (A) NMR-based PoC (red), (B) NIRS (blue), and (C) UV-Vis spectroscopy (grey) techniques assisted by supervised models (e.g., kNN, Logistic Regression, Naïve Bayes, Neural Network and Random Forest). The models were trained using the NMR-based traits (e.g., T_1, T_2 and A-ratio), NIRS (670nm peak) and UV-Vis (415nm peak) values of each sample. Power function fitting curves with confidence levels of 99% were used.

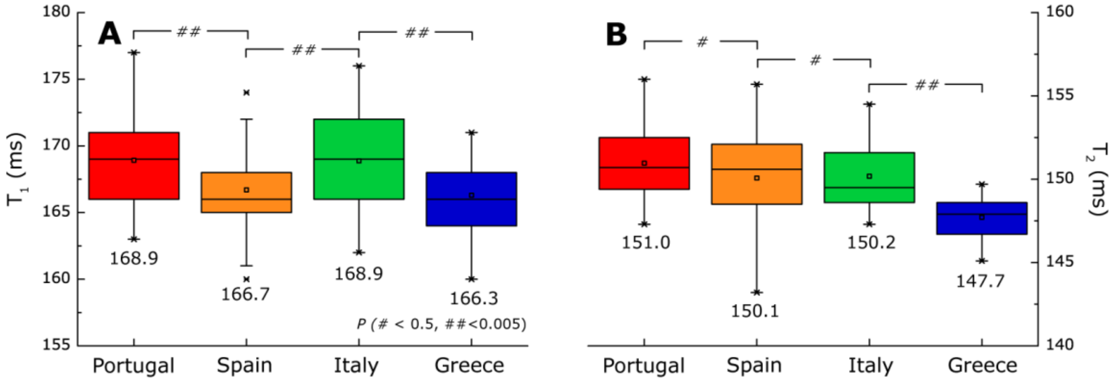

Supplementary Figure 3. Identification regions of origin for OO using NMR-based traits. (A) T_1 relaxation and (B) T_2 relaxation values obtained for the different commercial brands EVOOs originated from various countries (i.e., 3 Greece, 4 Italy, 9 Portugal, 5 Spain). The box plots represent 25% and 75% quantile of the entire measurements. One tailed Student's *t*-test was used to calculate the *P*-value.

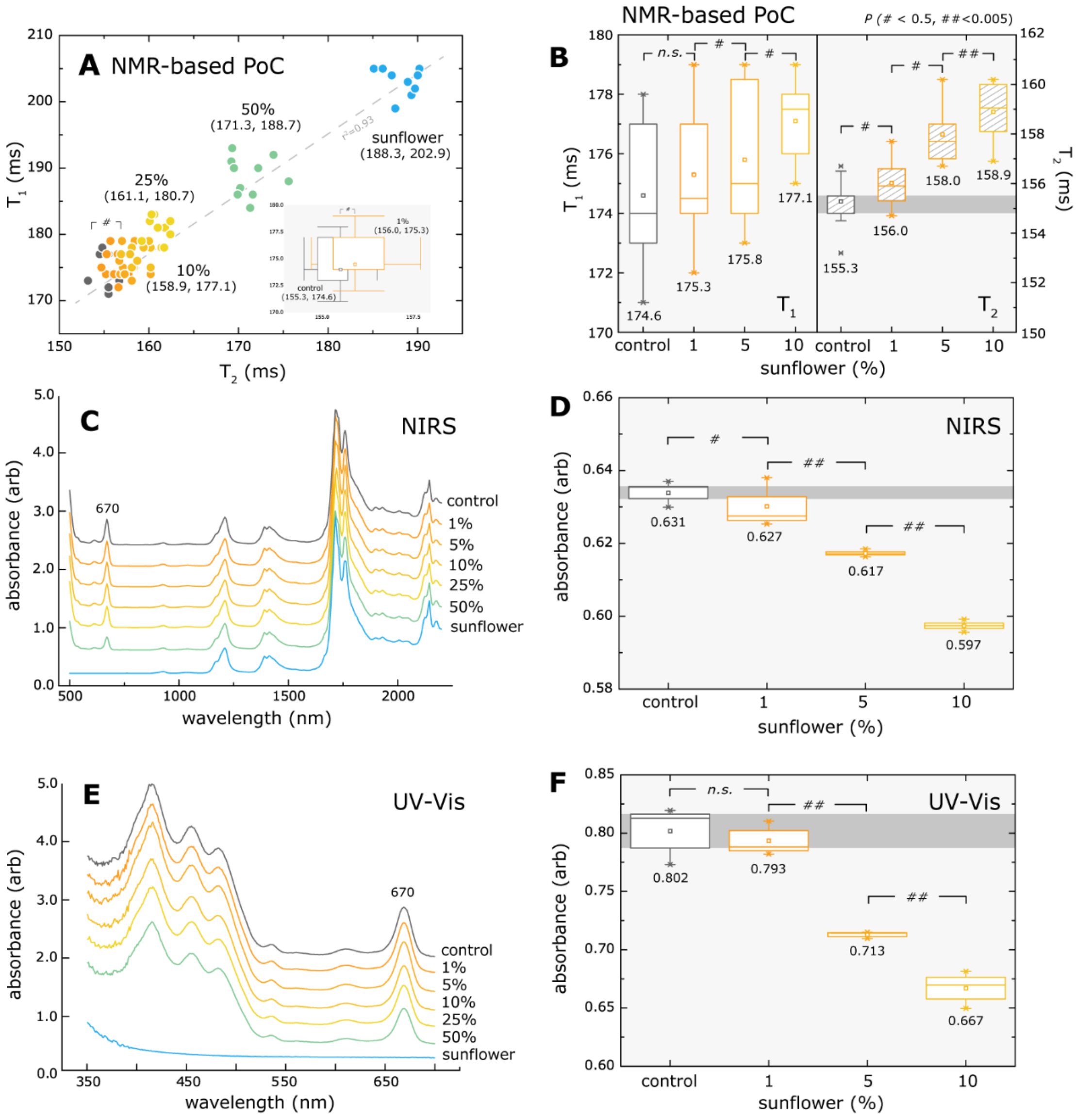

Supplementary Figure 4. Limit-of-detection (LOD) of the NMR-based traits versus state-of-the-art technologies (e.g., UV-Vis, NIR spectroscopy). The EVOOs (as control) were mixed with sunflower oil in concentration of 1%, 5%, 10%, 25%, and 50% to mimic the cases of adulteration. (A) Two-dimensional T_1-T_2 magnetic state diagram of the EVOOs (grey) as a function of sunflower oils concentration(coloured). The mean coordinates (T_2, T_1) were denoted for each dilution. Data points were linearly fitted (r^2=0.93) with function y=0.85x+42.13. The zoom-in plot indicates the OOs (as control) and OOs with 1% adulteration, the boxplots indicating 25% and 80% percentiles of the entire measurements. (B) The averaged T_1 (without strips, left) andT_2 (with strips, right) relaxations of the most overlapped region (e.g., control, 1%, 5% and 10%). (C) NIRS spectra taken from 500 nm to 2250 nm. (D) Multiple samplings were taken for each dilution. The maximum peak deviations were found to be at the 670 nm. (E) UV-Vis spectra taken from 250 nm to 700 nm. Multiple samplings were taken for each dilution. UV-Vis- The most significant peak was at 670 nm. (F)The box plots represent standard error of median quantile of the entire measurements. Two tailed Student's *t*-test was used to calculate the *P*-value. The LODs were 1%, 1%, and 5%, for NMR-based PoC, NIRS and UV-Vis, respectively.

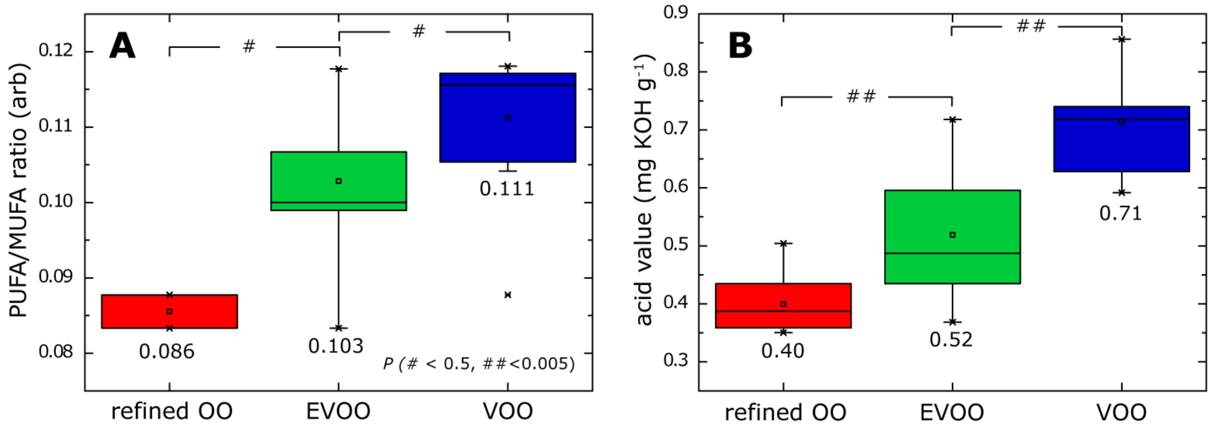

Supplementary Figure 5. The composite effect on molecular microenvironment contributes to the NMR-based phenotypic traits. (A) PUFA/MUFA ratio obtained from samples nutritional information (details in Supplementary Table 2), (B) Acid value obtained in this work (refer to Method). The boxplots represent standard error of median (A), and 25%, 75% (B) quantiles of the entire measurements. One tailed Student's *t*-test was used to calculate the *P*-value.

```
Tree reconstruction method - Neighbor Joining


TREE METRIC (ADDITIVE DISTANCE) MATRIX (AD)

Greece          0.000000 0.740000 0.845000 0.885000
Italy           0.740000 0.000000 0.645000 0.685000
Portugal        0.845000 0.645000 0.000000 0.690000
Spain           0.885000 0.685000 0.690000 0.000000


THE FOLLOWING STATISTICS ARE AVAILABLE FOR
A GIVEN DISSIMILARITY (D) AND AN OBTAINED TREE METRIC (AD)


Least-squares coefficient  Sum (Dij-ADij)^2  = 0.0081000000
                 i<j
Average absolute difference  Sum |Dij-ADij|/(n(n-1)/2)  = 0.0300000000
                 i<j
Maximum absolute difference   Max|Dij-ADij| = 0.0450000000
                 i,j
Total length of the tree   L  = 1.480000


        TREE EDGES WITH THEIR LENGTHS

        5--2          0.270000
        6--3          0.325000
        1--5          0.470000
        5--6          0.050000
        6--4          0.365000
```

Supplementary Figure 6. Construction of phylogenetic tree with NMR-based traits. Pair-wise comparison matrix[1] between region of origin (see Figure 3 in Main Text) was used as distance matrix for the construction of a phylogenetic tree (see Figure 4 in Main Text) using Neighbours-Joining algorithm[2].

kNN	EVOO	VOO	REF	Sum
**EVOO**	19	1	1	21
**VOO**	1	7	0	8
**REF**	1	0	6	7
Sum	21	8	7	**36**

L.R.	EVOO	VOO	REF	Sum
**EVOO**	19	1	1	21
**VOO**	1	7	0	8
**REF**	1	0	6	7
Sum	21	8	7	**36**

N.B.	EVOO	VOO	REF	Sum
**EVOO**	17	1	3	21
**VOO**	0	8	0	8
**REF**	1	0	6	7
Sum	18	9	9	**36**

N.N.	EVOO	VOO	REF	Sum
**EVOO**	19	1	1	21
**VOO**	1	7	0	8
**REF**	1	0	6	7
Sum	21	8	7	**36**

R.F.	EVOO	VOO	REF	Sum
**EVOO**	19	1	1	21
**VOO**	2	6	0	8
**REF**	2	0	5	7
Sum	23	7	6	**36**

(Rows: ACTUAL; columns: PREDICTED)

Supplementary Figure 7. The evaluation metrics of various supervised models for the classification of OOs (e.g., VOO, EVOO, refined OO). Supervised models were trained using the NMR-based traits (e.g., T_1,T_2 and A-ratio). The supervised models (e.g., kNN, Logistic regression (L.R.), Naive bayes (N.B.), Neural network (N.N.), and Random Forest (R.F.))[3] were used and validated using the leave-one-out method and inferred as two-dimensional confusion matrices (i.e., predicted versus actual classes).

kNN	Greece	Italy	Portugal	Spain	Sum
**Greece**	12	7	8	3	30
**Italy**	5	18	15	2	40
**Portugal**	5	15	57	13	90
**Spain**	5	6	13	26	50
Sum	27	46	93	44	**210**

(Rows: ACTUAL; columns: PREDICTED)

L.R.	Greece	Italy	Portugal	Spain	Sum
**Greece**	6	2	8	14	30
**Italy**	0	0	28	12	40
**Portugal**	0	0	74	16	90
**Spain**	10	0	29	11	50
Sum	16	2	139	53	**210**

(Rows: ACTUAL; columns: PREDICTED)

N.B.	Greece	Italy	Portugal	Spain	Sum
**Greece**	17	1	7	5	30
**Italy**	6	0	29	5	40
**Portugal**	8	2	60	20	90
**Spain**	10	1	25	14	50
Sum	41	4	121	44	**210**

(Rows: ACTUAL; columns: PREDICTED)

N.N.	Greece	Italy	Portugal	Spain	Sum
**Greece**	12	3	9	6	30
**Italy**	7	12	21	0	40
**Portugal**	5	6	71	8	90
**Spain**	5	2	17	26	50
Sum	29	23	118	40	**210**

(Rows: ACTUAL; columns: PREDICTED)

R.F.	Greece	Italy	Portugal	Spain	Sum
**Greece**	11	4	9	6	30
**Italy**	5	15	18	2	40
**Portugal**	7	16	55	12	90
**Spain**	3	4	19	24	50
Sum	26	39	101	44	**210**

(Rows: ACTUAL; columns: PREDICTED)

Supplementary Figure 8. The evaluation metrics of various supervised models for the regions of origins (e.g., Greece, Italy, Portugal, Spain) for OO. Supervised models were trained using the NMR-based traits (e.g., T_1, T_2 and A-ratio). The supervised models (e.g., kNN, Logistic regression (L.R.), Naive bayes (N.B.), Neural network (N.N.), and Random Forest (R.F.))[3] were used and validated using the leave-one-out method and inferred as two-dimensional confusion matrices (i.e., predicted versus actual classes).

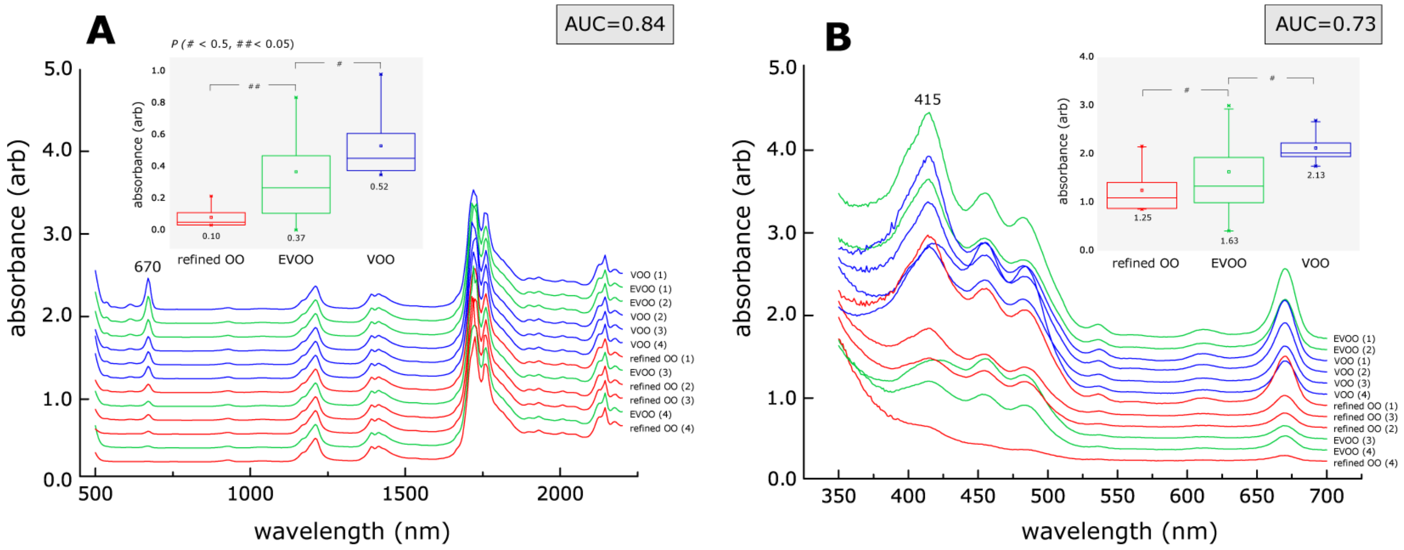

Supplementary Figure 9. Classification of OO using state-of-the-art technologies. Classification of OO by type (e.g., VOO, EVOO, refined OO) against the gold-standard techniques such as (A) NIRS, and (B) UV-Vis. Each curve represents the average of 3 measurements for each sample. The box plots represent the standard error of the median quantile of the entire measures. Sensitivity and specificity of each analysis were calculated using the AUC of the ROC curve (Table 1).

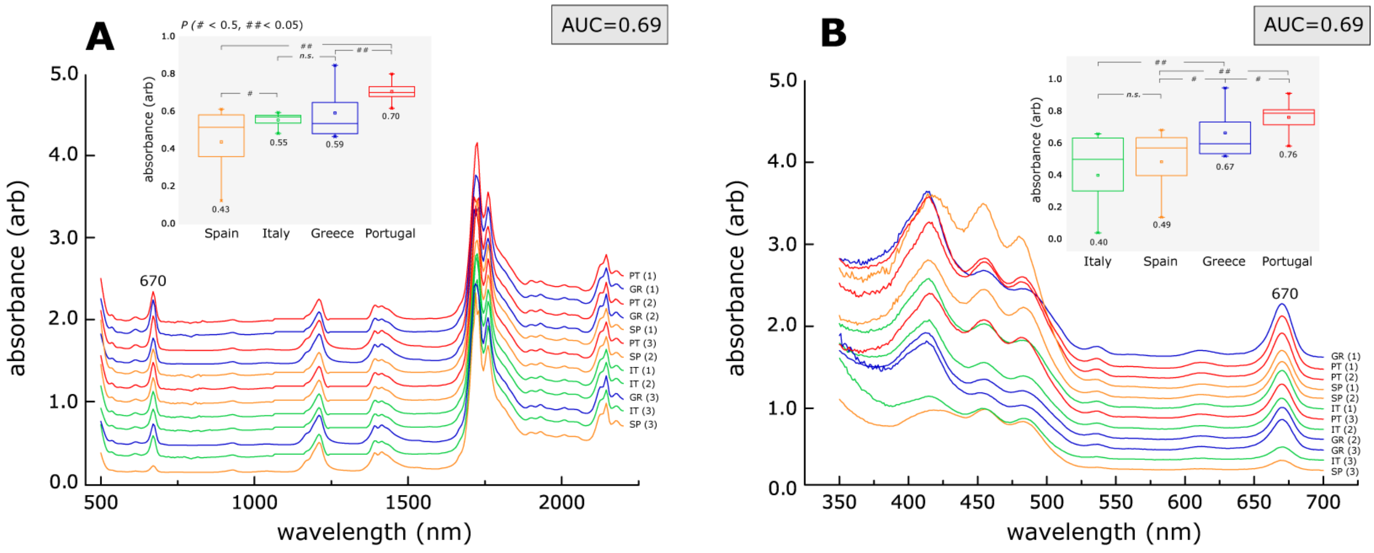

Supplementary Figure 10. Identification of the regions of origin for OO using state-of-the-art technologies. Various EVOO samples taken from various region of origin (i.e., Greece, Italy, Portugal, Spain). Of these, 3 samples were randomly chosen and measured by the gold-standard techniques. (A) NIRS, and (B) UV-Vis. Each curve represents the average of 3 measures for each sample. The box plots represent the standard error of the median quantile of the entire measures. Sensitivity and specificity of each analysis were calculated using the AUC of the ROC curve (Table 2).

Supplementary Table 1. The mean values for measurement of OOs using NMR-based PoC. The mean T_1, T_2 relaxation times, A-ratio, and obtained acid value for EVOOs, VOOs and refined OO with respect to their countries of origin. Undisclosed countries are denoted as not defined (n.d.).

Type	Region	T_1 (ms)	T_2 (ms)	A-ratio	Acid Value (mg KOH g^{-1})
EVOO	Greece	164.3	148.3	1.11	0.456
EVOO	Greece	167.1	146.2	1.14	0.578
EVOO	Greece	167.5	148.7	1.13	0.510
EVOO	Italy	167.8	148.7	1.13	0.671
EVOO	Italy	170.6	150.8	1.09	0.718
EVOO	Italy	171.5	153.1	1.12	0.414
EVOO	Italy	165.6	148.2	1.12	0.478
EVOO	Portugal	168.9	150.1	1.13	0.487
EVOO	Portugal	166.8	151.9	1.10	0.418
EVOO	Portugal	166.6	150.3	1.11	0.575
EVOO	Portugal	168.7	151.2	1.12	0.616
EVOO	Portugal	170.2	151.5	1.12	0.624
EVOO	Portugal	172.7	149.8	1.15	0.435
EVOO	Portugal	170.8	154.2	1.11	0.561
EVOO	Portugal	167.2	151.6	1.10	0.673
EVOO	Portugal	168.4	148.1	1.14	0.596
EVOO	Spain	165.2	152.1	1.09	0.409
EVOO	Spain	166.6	149.9	1.11	0.469
EVOO	Spain	166.8	153.1	1.09	0.391
EVOO	Spain	167.3	144.8	1.16	0.449
EVOO	Spain	167.6	150.6	1.14	0.368
\VOO	*n.d.*	174.4	153.3	1.14	0.740
VOO	Portugal	172.9	149.9	1.15	0.628
VOO	Portugal	173.3	152.7	1.14	0.740
VOO	Portugal	174.4	152.1	1.15	0.648
VOO	Portugal	173.5	152.2	1.14	0.718
VOO	Portugal	174.6	154.8	1.13	0.592
VOO	Portugal	174.6	155.3	1.13	0.787
VOO	Portugal	177.1	155.1	1.14	0.860
VOO+REF	Portugal	164.1	146.6	1.12	0.435
VOO+REF	Portugal	163.9	146.1	1.12	0.350
VOO+REF	Portugal	163.5	144.3	1.13	0.364
VOO+REF	Portugal	162.3	150.4	1.08	0.504
VOO+REF	Spain	160.3	144.2	1.11	0.359
VOO+REF	Spain	163.5	146.2	1.12	0.397
VOO+REF	Spain	161.9	146.0	1.11	0.387

Supplementary Table 2. The details of OO as disclosed by manufacturers. The nutritional information (e.g., fatty acid profiles, acidity) of each olive oils sample as disclosed by manufacturers. It is worth noting that the information is not scientifically cross-validated. Undisclosed countries are denotes as not defined (n.d.).

Type	Region	Manufacturer	Nutritional Information (per 100g)			
			SAFA (g)	MUFA (g)	PUFA (g)	Max acidity (%)
EVOO	Greece	Agric	14.0	77.0	9.0	0.8
EVOO	Greece	Omega live	13.6	70.7	7.3	0.8
EVOO	Greece	Molon	12.8	70.5	8.3	0.4
EVOO	Italy	Antika	-	-	-	-
EVOO	Italy	Costa'Oro	15.0	-	-	-
EVOO	Italy	Ewen	-	-	-	0.8
EVOO	Italy	Berio	13.9	70.4	7.0	0.6
EVOO	Portugal	GALLO - Colheira Madura	15.0	68.0	7.9	0.3
EVOO	Portugal	Oliveira da Serra - Gourmet	15.0	69.0	6.9	0.3
EVOO	Portugal	Herdade do Esporão - Azeite DOP	13.1	71.8	6.3	0.3
EVOO	Portugal	GALLO - Clássico	15.0	68.0	7.9	0.7
EVOO	Portugal	GALLO - Reserva	15.0	68.0	7.9	0.5
EVOO	Portugal	Vidigueira	13.1	-	-	0.8
EVOO	Portugal	Chaparro - Origens	13.3	-	-	0.7
EVOO	Portugal	Oliveira da Serra - Clássico	13.0	72.0	6.0	0.5
EVOO	Portugal	Flor do Alentejo	13.0	-	-	0.8
EVOO	Spain	Olivolia	14.0	79.0	7.0	0.5
EVOO	Spain	Froiz	13.0	-	-	-
EVOO	Spain	Mercadona	13.2	71.2	6.3	0.4
EVOO	Spain	Rego - Arbequina	-	-	-	-
EVOO	Spain	Rego	-	-	-	-
VOO	*n.d.*	Continente	13.1	-	-	2.0
VOO	Portugal	Oliveira da Serra - Versátil	14.5	68.6	8.1	0.9
VOO	Portugal	Oliveira da Serra - Virgem	15.0	69.0	8.1	0.9
VOO	Portugal	GALLO - Delicado	15.0	68.0	7.9	1.0
VOO	Portugal	5 Soldos - Casto	13.1	71.8	6.3	0.5
VOO	Portugal	Chaparro - Virgem	14.5	-	-	0.9
VOO	Portugal	Vila Branca	14.0	77.0	9.0	0.9
VOO	Portugal	Guia	12.0	-	-	0.7
VOO+REF	Portugal	5 Soldos - Azeite	13.1	71.8	6.3	1.0
VOO+REF	Portugal	Oliveira da Serra - Azeite	13.0	72.0	6.0	1.0
VOO+REF	Portugal	Serrata	14.0	-	-	1.0
VOO+REF	Portugal	Rustica	14.0	-	-	1.0
VOO+REF	Spain	Froiz	14.0	-	-	1.0
VOO+REF	Spain	Olearia del Olivar	11.9	-	-	1.0
VOO+REF	Spain	La Española	14.0	-	-	-

References

1. Ramík, J. Pairwise Comparison Matrices in Decision-Making. in *Pairwise Comparisons Method: Theory and Applications in Decision Making* (ed. Ramík, J.) 17–65 (Springer International Publishing, 2020). doi:10.1007/978-3-030-39891-0_2.
2. Saitou, N. & Nei, M. The neighbor-joining method: a new method for reconstructing phylogenetic trees. *Molecular Biology and Evolution* **4**, 406–425 (1987).
3. Demšar, J. *et al.* Orange: data mining toolbox in python. *J. Mach. Learn. Res.* **14**, 2349–2353 (2013).
